# Die Arbeitsbeziehung im Coaching: Ein Forschungsüberblick und Handlungsempfehlungen für die Praxis

**DOI:** 10.1007/s11613-022-00774-3

**Published:** 2022-07-20

**Authors:** Carolin Graßmann

**Affiliations:** VICTORIA International University of Applied Sciences, Bernburger Straße 24/25, 10963 Berlin, Deutschland

**Keywords:** Coaching, Arbeitsbeziehung, Beziehungsqualität, Coaching, Working alliance, Relationship quality

## Abstract

Eine gelingende Arbeitsbeziehung zwischen Coach und Coachee ist einer der zentralen Erfolgsfaktoren im Coaching und kann erklären, warum manche Coachingprozesse erfolgreicher verlaufen als andere. Dieser Beitrag liefert einen Überblick über den Stand der Forschung zur Arbeitsbeziehung im Coaching. Er zeigt die Bedeutung der Arbeitsbeziehung zu verschiedenen Outcome-Ergebnissen für Coachees auf sowie Faktoren, die die Arbeitsbeziehung fördern oder auch nicht fördern können. Darüber hinaus wird die Rolle der Arbeitsbeziehung im digitalen Coaching-Setting näher beleuchtet. Der Beitrag benennt offene Fragen für die Coaching-Forschung und leitet Handlungsempfehlungen für die Coaching-Praxis ab.

## Abstract (en)

The working alliance between coach and coachee is one of the key factors in coaching and can explain why some coaching processes are more successful than others. This paper provides an overview of the current state of research on the working alliance in coaching. It highlights the importance of the working alliance to various outcomes for coachees as well as factors that may or may not promote the working alliance. It also takes a closer look at the role of the working alliance in the digital coaching setting. This paper identifies open questions for coaching research and provides recommendations for coaching practice.

## Einleitung

Die Wirksamkeit von Coaching ist mittlerweile unbestritten. Es gibt zahlreiche Überblicksartikel (Theeboom et al. 2014; Jones et al. 2016; Wang et al. 2022), die empirisch zeigen können, dass Klienten nicht nur zufriedener werden und sich wohler fühlen, sondern auch ihre Ziele erreichen, besser mit Herausforderungen umgehen können und die Leistung sich erhöht. Die Wirksamkeit von Coaching scheint allerdings nicht für alle Coachees gleichermaßen zu erfolgen. Manche Coachingprozesse können für die Coachees sehr viel bewirken, aber andere Coachees profitieren weniger davon. Deshalb wird die Frage verstärkt aufgeworfen, wie man sich erklären kann, warum manche Coachingprozesse erfolgreicher verlaufen als andere (Theeboom et al. 2014). Diese Frage könnte in der Coachingpraxis dazu beitragen, den Erfolg des Coachings weiter zu steigern, indem konkrete Handlungsmöglichkeiten für Coaches, Coachees und Organisationen, die Coaching in Auftrag geben, abgeleitet werden.

Einer der prominentesten Erfolgsfaktoren in Coachingprozessen ist die Arbeitsbeziehung zwischen Coach und Coachee (Graßmann et al. 2020). Die Arbeitsbeziehung bezieht sich auf die Qualität des Engagements von Coach und Coachee in der kollaborativen, zielgerichteten Zusammenarbeit im Coaching (O’Broin und Palmer 2006). Sie wurde bereits früh als einer der zentralen Erfolgsfaktoren im Coaching diskutiert (z. B. Wasylyshyn 2003). Zahlreiche Forschungsarbeiten haben sich in den vergangenen Jahren diesem Thema empirisch gewidmet. Durch die Vielzahl der Forschungsarbeiten in diesem Bereich wird es immer komplexer und damit schwieriger, Schlussfolgerungen daraus zu ziehen. Viele dieser Artikel sind für die Coachingpraxis schwer zugänglich und wenig verständlich. Zudem erscheint der aktuelle Forschungsstand noch sehr fragmentiert und besteht oft aus einzelnen empirischen Zusammenhängen, die noch keinem gemeinsamen theoretischen Grundgerüst folgen. Dieser Beitrag soll daher einen deutschsprachigen Überblick über den aktuellen Stand der Forschung zur Arbeitsbeziehung zwischen Coach und Coachee bieten. Er beleuchtet zunächst die Bedeutung der Arbeitsbeziehung im Coaching, wie sie gefördert werden kann und inwiefern die Arbeitsbeziehung im digitalen Setting zum Tragen kommt. Er zeigt zudem weiterhin offene Fragestellungen auf und versucht, Handlungsempfehlungen für die Coaching-Praxis abzuleiten.

## Das Konzept der Arbeitsbeziehung

Das Konzept der Arbeitsbeziehung stammt ursprünglich aus dem Bereich der Psychotherapie. Es lässt sich bis zu Freuds Schriften zurückverfolgen (für eine Beschreibung der Geschichte der Arbeitsbeziehung in der Psychotherapie vgl. Horvath et al. 2011). Später wurde die Arbeitsbeziehung als theorieübergreifendes Konzept über psychoanalytische Ansätze hinaus erweitert und als ein gemeinsamer Erfolgsfaktor für alle therapeutischen Ansätze positioniert (Rosenzweig 1936). Bordin (1979) schlug weiterhin vor, dass die förderliche Funktion der Arbeitsbeziehung für alle helfenden Professionen verallgemeinert werden kann und nicht nur auf die Psychotherapie beschränkt ist. Er ging davon aus, dass die Arbeitsbeziehung überall dort einen Schlüssel, wenn nicht sogar den entscheidenden Schlüssel zum Erfolg darstellt, wo eine Person in einem Veränderungsprozess durch eine andere Person begleitet wird. Übereinstimmend mit seiner These zeigten im Laufe der Zeit zahlreiche Studien auch in anderen helfenden Professionen die Bedeutung der Arbeitsbeziehung für die Ergebnisse des Veränderungsprozesses – nicht nur in der Psychotherapie (vgl. Horvath et al. 2011; Martin et al. 2000), sondern auch im Mentoring (Eby et al. 2013), der Supervision (Ramos-Sánchez et al. 2002) und der individuellen Karriereberatung (Milot-Lapointe et al. 2021). Dies unterstreicht die Schlüsselrolle der Arbeitsbeziehung in professionellen zwischenmenschlichen Interaktionen.

**Figure 1:**
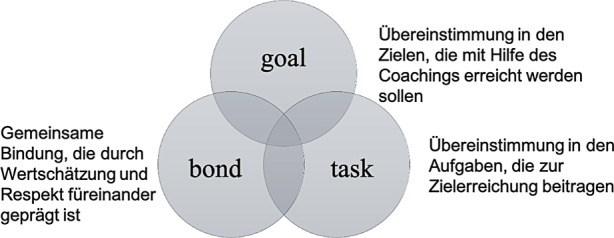

Es besteht jedoch noch eine gewisse Unklarheit hinsichtlich der Bezeichnung des Konzepts, was sich in der Verwendung unterschiedlicher Begriffe widerspiegelt, wie etwa „Coachingbeziehung“, „Beziehungsqualität“ oder „Allianz“. In der englischsprachigen Literatur hat sich mittlerweile vor allem „working alliance“ als Begriff durchgesetzt. Diese lässt sich in drei Dimensionen unterteilen (Bordin 1979; Horvath und Greenberg 1989; vgl. Abb. 1): Übereinstimmung in den Zielen, die im Coaching verfolgt werden sollen („goal“), Übereinstimmung darin, welche Aufgaben für die Zielerreichung notwendig sind („task“), und eine gemeinsame Bindung, die durch Vertrauen, Respekt und Sympathie füreinander geprägt ist („bond“). So wird eine starke Arbeitsbeziehung oftmals darüber erfasst, dass Coachees und Coaches der Auffassung sind, dass sie auf Ziele hinarbeiten, über die sie sich einig sind, darüber einstimmen, woran es wichtig ist zu arbeiten, und sie glauben, dass das Coaching helfen wird, diese Ziele zu erreichen. Die Bindung, die sie miteinander eingehen, wird in der Regel über den subjektiven Eindruck der Coachees und Coaches erfasst, inwieweit sie sich mögen, sich gegenseitig schätzen und zueinanderstehen, auch wenn sie jeweils andere Ansichten vertreten.

O’Broin und Palmer (2006) übertrugen das Konzept der Arbeitsbeziehung dann explizit auf den Coaching-Kontext und argumentierten, dass sie sich sehr wahrscheinlich als förderlich für Coaching-Ergebnisse erweist. Dennoch gab es erst in den vergangenen Jahren ausreichend empirische Studien, die dieser Fragestellung nachgingen.

## Bedeutung der Arbeitsbeziehung im Coaching

Um die Bedeutung der Arbeitsbeziehung im Coaching näher zu beleuchten, wird im Folgenden zunächst beschrieben, mit welchen Ergebnissen für Coachees sie in Zusammenhang gebracht werden kann. Es werden offene Fragen und Handlungsempfehlungen abgeleitet.

### Breites Wirkungsspektrum der Arbeitsbeziehung

Die ausreichend große Anzahl an Studien zur Arbeitsbeziehung im Coaching machte deren quantitative Zusammenfassung mittlerweile möglich. Graßmann et al. (2020) haben hierfür systematisch alle vorhergehenden Studien gesucht, die den Zusammenhang zwischen der Beziehungsqualität im Coaching und den möglichen Ergebnissen für Coachees quantitativ untersuchten. Sie identifizierten 27 Studien, die in die weitere Analyse aufgenommen werden konnten. Sie berücksichtigten hierbei verschiedene Ergebniskategorien: affektive Ergebnisse, kognitive Ergebnisse, individuelle Ergebnisse sowie unbeabsichtigte negative Nebenwirkungen, die durch das Coaching verursacht werden.

Diese Studie zeigte für alle Ergebniskategorien signifikante Zusammenhänge auf, mit den stärksten Zusammenhängen für die affektiven Ergebnisse für Coachees, insbesondere für die Zufriedenheit des Coachees mit dem Coaching, die wahrgenommene Effektivität des Coachings, aber auch die Selbstwirksamkeit des Coachees. Dass die Qualität der Arbeitsbeziehung am stärksten mit der Zufriedenheit der Coachees mit dem Coaching insgesamt und auch dem Coachingerfolg zusammenhängt, scheint nicht ungewöhnlich zu sein. So lässt sich dieses Muster z. B. auch in der individuellen Karriereberatung (Milot-Lapointe et al. 2021) finden. Dies könnte daran liegen, dass Coachees die Arbeitsbeziehung als Indikator nutzen, um die Qualität der Intervention insgesamt zu bewerten (ebd.). Dies sollte sich auch auf die Weiterempfehlung und Reputation der Coaches auswirken, auch wenn dies noch nicht empirisch gezeigt wurde.

Die Arbeitsbeziehung hängt auch vergleichsweise stark mit den kognitiven Ergebnissen für Coachees zusammen, wie z. B. der Selbstreflexion. Geringere, aber immer noch mittelstarke Zusammenhänge zeigten sich zu den individuellen Ergebnissen der Coachees, vor allem der Zielerreichung sowie der Vermeidung von negativen Nebenwirkungen. Insgesamt zeigt sich ein empirisch robuster Zusammenhang zwischen der Arbeitsbeziehung und einem breit gefächerten Wirkungsspektrum für Coachees. Damit reiht sich Coaching in die Erkenntnisse aus anderen helfenden Professionen ein und bestätigt damit Bordins (1979) Annahme, dass es sich bei der Arbeitsbeziehung um einen wichtigen Schlüssel zum Erfolg von begleiteten Veränderungsprozessen handelt.

Neben diesen Zusammenhängen wurden aber auch weitere Auswirkungen untersucht, so z. B., ob eine geringe Qualität der Arbeitsbeziehung mit einem Coachingabbruch in Verbindung steht (s. Schermuly 2018). Coachingabbruch wurde hierbei so definiert, dass das Coaching vor der vereinbarten Zeit durch den oder die Coachee beendet wurde, ohne dass sich der Coachingerfolg bereits eingestellt hat (ebd.). In den Coachingprozessen, die durch die Coachees abgebrochen wurden, bewerteten Coaches die Arbeitsbeziehung als weniger stark als in den Coachingprozessen, die nicht abgebrochen wurden. Dies ist ein erster Hinweis darauf, dass eine gute Arbeitsbeziehung mit weniger Coachingabbrüchen einhergeht, auch wenn hierzu weitere Studien nötig sind.

Darüber hinaus scheint der Zusammenhang zwischen der Arbeitsbeziehung und den Wirkungen für Coachees heterogen gestaltet zu sein. Das bedeutet, dass die Arbeitsbeziehung in manchen Coaching-Prozessen sehr stark zum Tragen kommt und in anderen Coaching-Prozessen sich weniger stark auf den Coachingerfolg auswirkt. Woran das liegt, lässt sich noch nicht gut erklären. Zumindest an der Länge des Coachings, an der Zielgruppe (Studierende oder nicht) oder an der Expertise der Coaches (frisch ausgebildete Coaches oder Coaches mit mehr Erfahrung) scheint es erst einmal nicht zu liegen (Graßmann et al. 2020).

### Die Wahl der Perspektive

Obwohl die Arbeitsbeziehung erst zwischen den beiden Akteuren Coach und Coachee entsteht, müssen beide die Arbeitsbeziehung nicht identisch wahrnehmen und einschätzen (Baron et al. 2011; Gessnitzer und Kauffeld 2015). Dies ist kein ungewöhnliches Bild und lässt sich auch in anderen Professionen finden: Auch in der Psychotherapie weichen Klient:innen von der Einschätzung ihrer Psychotherapeut:innen ab (Kivlighan et al. 2017). Und auch in der Führung ist es nicht ungewöhnlich, dass Führungskräfte und ihre Mitarbeitenden ihre Beziehung zueinander unterschiedlich einschätzen (Gerstner und Day 1997).

Die Einschätzung der Arbeitsbeziehung durch Coachees scheint den Coachingerfolg besser vorherzusagen als die Einschätzung durch Coaches (Graßmann et al. 2020), weil die Wahrnehmung der Coachees ausschlaggebend dafür ist, wie viel sie über sich preisgeben wollen und wie sehr sie die Veränderungen, die im Coaching besprochen werden, anstreben und umsetzen wollen. Diese Annahme lässt sich mit sehr viel größerer empirischer Basis im Bereich der Psychotherapie stützen, wo die Einschätzungen der Arbeitsbeziehung durch die Klient:innen die Psychotherapie-Ergebnisse besser vorhersagten als die Einschätzung durch die Psychotherapeut:innen (z. B. Zilcha-Mano & Errázuriz 2015).

### Offene Fragen zur Bedeutung der Arbeitsbeziehung im Coaching

Eine große Einschränkung zu den oben aufgeführten Studien ist, dass keine Kausalität abgeleitet werden kann. Es ist zwar theoretisch anzunehmen, dass eine stärkere Arbeitsbeziehung zu mehr Coachingerfolg führt. Es kann aber auch möglich sein, dass Coachees in erfolgreichen Coachingprozessen aufgrund des Erfolgs auch die Arbeitsbeziehung zum Coach besser einschätzen. Dies würde einem Halo-Effekt gleichen (Horvath et al. 2011), bei dem der Zusammenhang zwischen der Arbeitsbeziehung und dem Coachingerfolg überinterpretiert wird, weil beides von derselben Person eingeschätzt wird. Eine weitere Möglichkeit ist zudem, dass die Arbeitsbeziehung sich nicht auf den Coachingerfolg auswirkt, sondern vielmehr die Erwartungen und die Bereitschaft der Coachees zu Beginn des Coachings reflektiert. So fanden de Haan et al. (2020), dass die Arbeitsbeziehung mit Coachingerfolg nach der ersten Coachingsitzung zusammenhängt, aber nicht mehr mit der Steigerung des Coachingerfolgs im Laufe des Coachingprozesses. Sie vertreten daher die Ansicht, dass die Arbeitsbeziehung einen Klienten-Faktor und nicht einen relationalen Faktor darstellt, der zwischen Coach und Coachee entsteht. Im Bereich der Psychotherapie geht man von einem Mittelweg zwischen beiden Annahmen aus: Ein Teil des Zusammenhangs zwischen Arbeitsbeziehung und Interventionserfolg geht darauf zurück, inwiefern die Klient:innen generell fähig sind, eine hilfreiche Arbeitsbeziehung einzugehen, und der andere Teil des Zusammenhangs lässt sich durch die eigentliche dyadische Beziehung erklären (Zilcha-Mano 2017). Dies ist eine interessante Fragestellung, und weitere Studien im Bereich Coaching sind notwendig, um herauszufinden, wie und warum sich die Rolle der Arbeitsbeziehung im Laufe des Coaching-Prozesses ändert und welchen Anteil die Fähigkeit der Coachees, Arbeitsbeziehungen generell einzugehen, daran hat.

Neben der zeitlichen Komponente scheint die Arbeitsbeziehung auch nicht in allen Coachingprozessen gleichermaßen mit dem Coachingerfolg zusammenzuhängen (z. T. ist auch gar kein Zusammenhang ersichtlich, z. B. bei Busch et al. 2021). Hierbei bleibt die Frage offen, wann sie eine bedeutende Rolle einnimmt und wann sie durch andere Bestandteile und Erfolgsfaktoren im Coaching möglicherweise ersetzt oder in ihrer Rolle minimiert wird. So lässt sich mutmaßen, dass es manchen Coachees schwerer fällt, sich auf den Coaching-Prozess einzulassen und sich zu öffnen. Extraversion der Coachees hat sich z. B. als förderlich für den wahrgenommenen Coachingerfolg gezeigt (Jones et al. 2014). Dies könnte zur Folge haben, dass bei diesen Coachees eine starke Arbeitsbeziehung den Coachingerfolg stärker beeinflusst, weil es ihnen dadurch leichter fällt, sich zu öffnen und den Coaching-Prozess voranzubringen. Daneben könnte die Arbeitsbeziehung bei sensiblen Themen einen stärkeren Einfluss auf den Coachingerfolg haben als bei Themen, die eher an der Oberfläche verbleiben. Gerade bei den sensiblen Themen, bei denen Coachees nicht so leichtfertig etwas über sich preisgeben wollen, könnte eine starke Arbeitsbeziehung den Prozess besonders unterstützen.

### Empfehlungen für die Coaching-Praxis

Obwohl die Wirkrichtung unklar bleibt, ist zumindest zu Beginn des Coachingprozesses ein robuster Zusammenhang zwischen der Arbeitsbeziehung und den Ergebnissen für Coachees zu sehen. Daher können Coaches das Konzept der Arbeitsbeziehung als einen Indikator für die Qualität der gemeinsamen Arbeit mit den Coachees benutzen und als Ausgangsbasis für die eigene Reflexion verwenden. Vor allem die Unterscheidung der drei Dimensionen *goal, task* und *bond* könnte sich hierfür als nützlich erweisen, weil auf diese Weise konkrete Punkte für die weitere Reflexion abgeleitet werden können. Auch in der Supervision könnte sich das Besprechen der Arbeitsbeziehung und ihrer drei Komponenten als nützlich erweisen. Zudem sollten Coaches auch direkt die Einschätzung der Coachees erfragen, um sie mit der eigenen Einschätzung der Arbeitsbeziehung abzugleichen.

## Fördern der Arbeitsbeziehung

O’Broin und Palmer (2006) gehen davon aus, dass die Arbeitsbeziehung im Coaching nicht nur zu Beginn, sondern auch im Laufe des gesamten Coachingprozesses immer wieder neu verhandelt wird. Da der Zusammenhang zwischen der Arbeitsbeziehung und dem Coachingerfolg robust gezeigt werden kann, stellt sich die Frage, welche Faktoren die Arbeitsbeziehung stärken können.

### Faktoren, die die Arbeitsbeziehung (nicht) fördern können

Ebenso wie bei der Bedeutung der Arbeitsbeziehung für den Coachingerfolg existiert auch eine systematische Literaturübersicht zu den Faktoren, die den Aufbau der Arbeitsbeziehung begünstigen können (Graßmann und Schermuly 2020). Diese Arbeit fasste systematisch alle Studien zu dieser Fragestellung zusammen und integrierte so die Erkenntnisse aus insgesamt 18 Studien. Die Studienlage erscheint hierbei als sehr heterogen und zeigt eine Vielzahl unterschiedlicher Faktoren, die bisher betrachtet wurden. Im Folgenden werden zunächst die Faktoren auf der Seite der Coachees betrachtet, dann die Faktoren auf der Seite der Coaches und abschließend die Faktoren, die aus der Interaktion beider Seiten entstehen.

In dieser Überblicksstudie kristallisierte sich auf der Seite der Coachees insbesondere die Coachingmotivation als Faktor heraus, der mit einer stärkeren Arbeitsbeziehung einherging. Die Motivation bezieht sich hierbei auf die Bereitschaft der Coachees, Zeit und Energie zu investieren und sich zu verändern, und ist nicht nur für die Arbeitsbeziehung von Interesse, sondern auch für den späteren Coachingerfolg (Bozer und Jones 2018). Wenige Studien bezogen sich auf konkretes Verhalten der Coachees, konnten aber einzelne Verhaltensweisen aufzeigen. So fanden Ianiro und Kauffeld (2014), dass dominant-freundliches Verhalten der Coachees eine stärkere Arbeitsbeziehung vorhersagen konnte. Darüber hinaus scheint die Persönlichkeit der Coachees insgesamt betrachtet wenig zur Erklärung der Stärke der Arbeitsbeziehung beizutragen (z. B. de Haan et al. 2016). Persönlichkeit bezieht sich hierbei auf relativ stabile Eigenschaften der Coachees, wie z. B. Extraversion und Offenheit für neue Erfahrungen. Die Studien berücksichtigen hierbei zwar eine Vielzahl unterschiedlicher Persönlichkeitseigenschaften in diesem Sinne, unterschieden aber nicht nach dem zugrunde liegenden Coachingverständnis. Dennoch lässt sich bislang wenig bis kaum Evidenz dafür finden.

Auf der Seite des Coachs zeichnete sich insbesondere die wahrgenommene Kompetenz des Coachs als einflussreicher Faktor für die Arbeitsbeziehung ab (Graßmann und Schermuly 2020). Diese wahrgenommene Kompetenz bezieht sich auf die wahrgenommene Passung der Kompetenzen des Coachs zu dem, was sich die Coachees für ihr Anliegen wünschen (Boyce et al. 2010), bzw. inwiefern sich die Coaches selbst hierfür als kompetent einschätzen. Die zugrunde liegenden Studien bezogen sich auf die ganzheitlich eingeschätzte, subjektive Wahrnehmung von Kompetenz und nicht auf fachliche Hintergründe oder Methodenkenntnisse. Die Ergebnisse zeigen, dass die wahrgenommene Kompetenz des Coachs deutlich mit der Stärke der Arbeitsbeziehung zusammenhängt (Graßmann und Schermuly 2020). Die tatsächliche Erfahrung als Coach in Jahren scheint dagegen nicht mit ihr zusammenzuhängen. Auch in Bezug auf das Verhalten des Coachs zeigten sich etliche einzelne Verhaltensweisen als förderlich für die Arbeitsbeziehung zum Coachee. So fanden z. B. Henriques et al. (2017), dass Stimulation, Planung und die Strukturierung von Aktivitäten mit einer stärkeren Arbeitsbeziehung zusammenhingen. Auch dominant-freundliches Verhalten zeigte sich wieder auf der Seite des Coachs als förderlich für die Arbeitsbeziehung (Ianiro et al. 2013; Ianiro und Kauffeld 2014). Obwohl etliche Verhaltensweisen (oftmals einmalig und ohne Verhaltensbeobachtung) als förderlich gezeigt worden sind, fehlt bislang noch ein einheitlicher Rahmen, entlang dessen man diese theoretisch und praktisch nutzbar machen kann. Ebenso wie bei den Coachees erwies sich die Persönlichkeit der Coaches als weitestgehend uninteressant in Bezug auf die Arbeitsbeziehung (z. B. de Haan et al. 2016).

Abschließend können auch Faktoren, die erst durch die Interaktion der Charakteristiken von Coachee und Coach entstehen, zum Aufbau der Arbeitsbeziehung beitragen. Hieraus ergibt sich vor allem die Frage, ob ein Matching und die Ähnlichkeit der beiden Akteure zum Aufbau der Arbeitsbeziehung beitragen. Insgesamt betrachtet scheint allerdings weder die Ähnlichkeit in der Persönlichkeit noch ein Matching aufgrund dieser Eigenschaften mit einer stärkeren Arbeitsbeziehung in Verbindung zu stehen. Andererseits gibt es zunächst erste Hinweise, dass zumindest eine Ähnlichkeit im Verhalten für den Aufbau der Arbeitsbeziehung zuträglich ist. So konnten Ianiro et al. (2013) zeigen, dass wenn Coaches die Dominanz und die Freundlichkeit ihrer Coachees spiegelten, die Arbeitsbeziehung positiv damit in Zusammenhang stand.

### Brüche in der Arbeitsbeziehung

Arbeitsbeziehungen müssen im Laufe des Coaching-Prozesses nicht immer nur stärker werden, sondern können sich auch verschlechtern. Solche Brüche in der Arbeitsbeziehung sind bereits aus der Psychotherapie bekannt und lassen sich konzeptionell auch auf das Coaching übertragen (für eine detailliertere Darstellung vgl. Ehrenthal et al. 2020). In der Psychotherapie scheinen Brüche in der Arbeitsbeziehung oft aufzutreten, können aber auch wieder repariert werden und sogar zu einem stärkeren Therapieerfolg beitragen (Eubanks et al. 2018; Safran et al. 2011). Auch im Coaching kann es zu Brüchen der Arbeitsbeziehung kommen, auch wenn sie seltener sein mögen als in der Psychotherapie; wichtig ist, diese zu erkennen und gezielt zu reparieren (Ehrenthal et al. 2020). Bisher wurden Brüche der Arbeitsbeziehung im Coaching noch wenig empirisch untersucht, sondern eher der Umgang mit schwierigen Situationen im Coaching (für eine detaillierte Darstellung vgl. Möller und Zimmermann 2020), zu denen sie durch ihr Auftreten sicherlich beitragen können.

### Offene Fragen zur Förderung der Arbeitsbeziehung

Trotz der vereinzelten Studien zu dieser Fragestellung bleibt es weiterhin in weiten Teilen nicht vollständig geklärt, wie die Arbeitsbeziehung im Coaching konkret gefördert und aufrechterhalten werden kann. Die Motivation des Coachees und die wahrgenommene Kompetenz des Coachs scheinen unter den untersuchten Faktoren den deutlichsten Zusammenhang zur Arbeitsbeziehung aufzuweisen. Hier sind weitere Arbeiten nötig, die diese Zusammenhänge auch experimentell zeigen können. Auch der umgekehrte Effekt ist durchaus denkbar, bei dem sich Coachees durch eine starke Arbeitsbeziehung motivierter fühlen, an sich zu arbeiten, und auch der Coach sich hierdurch als kompetenter für das Anliegen des Coachees wahrnimmt bzw. von ihm oder ihr wahrgenommen wird. Da insbesondere die konkreten Verhaltensweisen oftmals nur einmalig untersucht und in der Regel lediglich mit Fragebögen erhoben wurden, ist weiterhin unklar, welche Verhaltensweisen zu einer starken Arbeitsbeziehung beitragen. Vor allem das Verhalten des Coachs erscheint hier interessant, da sich hieraus konkrete Handlungsempfehlungen für die Coaching-Praxis ableiten ließen. Erfreulich ist zumindest die Feststellung, dass ausgewählte Verhaltensweisen sich überhaupt auf die Arbeitsbeziehung auszuwirken scheinen, sodass die Arbeitsbeziehung auch veränderbar zu sein scheint. Zukünftig sollte weiter geklärt werden, was Coaches konkret tun (oder nicht tun) können, um die Arbeitsbeziehung aufzubauen oder aufrechtzuerhalten. Fast gar nicht beleuchtet wurde zudem die Rolle der Organisation der Coachees. Die Organisation kann durchaus einen Einfluss auf die Gestaltung der Arbeitsbeziehung nehmen (Lai und Smith 2021), und auch ein indirekter Einfluss ist möglich. So zeigte Cope (2021), dass die Vertrautheit der Coaches mit der Organisation der Coachees mit einer stärkeren Arbeitsbeziehung einherging.

### Empfehlungen für die Coaching-Praxis

Für Coaches lässt sich aus den Ergebnissen ableiten, dass die Arbeit an und mit der Motivation der Coachees eine nennenswerte Rolle spielt, sowohl für die Arbeitsbeziehung als auch für den Coachingerfolg. Für Organisationen lässt sich daraus ableiten, dass Mitarbeitende vor allem dann Zugang zu Coaching erhalten sollten, wenn sie auch die Bereitschaft zur Veränderung mitbringen. In Bezug auf die Bedeutung der wahrgenommenen Kompetenz sollten Coaches und Coachees auf die geeignete Passung für das Anliegen der Coachees achten, um die Arbeitsbeziehung bestmöglich zu unterstützen. Organisationen können hierfür einen großen Pool an möglichen Coaches nutzen, um dies zu gewährleisten. Da Erfahrung als Coach in Jahren sich per se weder als förderlich noch als hinderlich erwies, ist dies ein gutes Zeichen für Coaching-Novizen, die frisch in die Coaching-Praxis starten.

## Arbeitsbeziehungen im digitalen Setting

Nicht erst seit der Covid-19 Pandemie stellt die Digitalisierung einen der wichtigsten Mega-Trends dar, die in sämtlichen gesellschaftlichen und wirtschaftlichen Bereichen Einzug gehalten hat. Da sie die Arbeitswelt der Coachees nachhaltig verändert, ist es nicht verwunderlich, dass die Digitalisierung auch im Coaching an Bedeutung gewinnt. Eine Delphi-Studie zur Zukunft des Coachings im deutschsprachigen Raum (Schermuly et al. 2021) konnte hierbei zahlreiche Szenarien aufzeigen, die mit der Digitalisierung des Coachings zusammenhängen. Die Szenarien entstanden zunächst auf Basis einer Expertenbefragung und wurden dann in zwei Befragungswellen von mehreren Hundert Coaching-Akteuren hinsichtlich ihrer aktuellen und zukünftigen Verbreitung sowie ihrer Erwünschtheit eingeschätzt. Die digitalen Szenarien, die sich für die Zukunft des Coachings ableiten ließen, umfassten die Nutzung von virtuellen Umgebungen oder auch von Avataren, die Anwendung von Künstlicher Intelligenz in Form von Assistenzsystemen, die zunehmende Verbreitung von Coaching-Plattformen und von rein online durchgeführten Coaching-Weiterbildungen. Es zeigt sich in dieser Delphi-Studie insgesamt, dass die zunehmende Digitalisierung von Coaching zwar erwartet, aber nicht unbedingt auch als positiv betrachtet wird (ebd.).

### Coaching mit digitalen Kommunikationsmedien

Ob Coaching mit digitalen Kommunikationsmedien oder in Präsenz durchgeführt wird, sollte theoretisch einen Unterschied beim Coachingerfolg erwarten lassen. Die Media Richness Theory (Daft und Lengel 1986) nimmt an, dass die Wahl des Mediums davon abhängt, wie reichhaltig die Informationen sind, die man vermitteln möchte. Angenommen, dass die Informationen, die im Coaching ausgetauscht werden, sehr reichhaltig sind, ließe sich schlussfolgern, dass in Präsenz die größte Wirkung erzielt werden sollte, weil hierüber die meisten Informationen vermittelt werden können. Etwas schlechter sollte digital durchgeführtes Coaching abschneiden, da hier weniger Informationen als in Präsenz vermittelt werden können, wie z. B. durch Körpersprache oder Bewegungen im Raum. Die empirische Evidenz zeigt allerdings keinen signifikanten Unterschied zwischen Präsenz- und Blended Coaching, bei dem digital vermittelte Anteile per Text oder Video im Laufe des Coachingprozesses integriert sind (Jones et al. 2016). Hierbei kann argumentiert werden, dass Coach und Coachee zumindest in Teilen immer noch in Präsenz zusammengearbeitet haben, was erklärt, warum kein Unterschied gefunden wurde. Passarelli et al. (2020) untersuchten Coachingprozesse, die ausschließlich in Präsenz oder per Videokonferenz-Software standfanden, und fanden ebenfalls keinen signifikanten Unterschied zwischen den beiden Formaten – weder wenn Coachees die Arbeitsbeziehung einschätzten noch wenn Coaches angaben, inwieweit die Coachees ihnen vertrauen würden. Zwar hatte das Medium selbst keinen direkten Einfluss auf die Arbeitsbeziehung, übte aber zumindest einen indirekten Einfluss über die wahrgenommene Reichhaltigkeit des genutzten Mediums aus (Passarelli et al. 2020). Dies deutet darauf hin, dass es wider Erwarten doch keinen oder nur einen kleinen indirekten Einfluss auf die Arbeitsbeziehung hat, ob Coach und Coachee in Präsenz, mit Hilfe von digitalen Kommunikationsmedien oder ausschließlich per Videokonferenz-Software miteinander arbeiten. Der Stand der Forschung ist allerdings noch sehr jung, wodurch robuste und differenzierte Schlussfolgerungen erschwert werden.

### Coaching durch Künstliche Intelligenz

Einen Schritt weiter im Sinne der Digitalisierung geht das Coaching durch Künstliche Intelligenz, bei dem kein menschlicher Coach mehr involviert ist, sondern der Coachee mit einem künstlichen Assistenten arbeitet (für eine Einführung zu Coaching durch Künstliche Intelligenz vgl. Graßmann und Schermuly 2021; Terblanche 2020). Oftmals werden hierfür Chatbots verwendet, die Coachees zeit- und ortsunabhängig in Anspruch nehmen können. Erste Studien zeigen, dass Coaching mit Hilfe von Künstlicher Intelligenz sehr wohl effektiv sein kann (Mai et al. 2021, 2022). Dies fügt sich nahtlos in die Forschung im Bereich *e‑mental health* ein, bei der bereits vielfach gezeigt werden konnte, dass diese Systeme ebenso gut zur Symptomlinderung beitragen können wie traditionelle Vorgehensweisen (Andrews et al. 2010; Merry et al. 2012).

Aufgrund der empirischen Evidenz sowie der prognostizierten zukünftig stärkeren Verbreitung im Coaching (Schermuly et al. 2021) stellt sich die Frage, welche Rolle die Arbeitsbeziehung noch spielt, wenn nur noch ein einziger menschlicher Akteur im Coaching verbleibt. Es erscheint kontraintuitiv, dass Coachees eine Arbeitsbeziehung zu einem nicht-menschlichen virtuellen Agenten aufbauen können, vor allem wenn die affektive Bindung zwischen Coach und Coachee betrachtet wird. Überraschenderweise hat die Forschung gezeigt, dass Personen sehr wohl eine Bindung zu einem virtuellen Agenten aufbauen können und diese Bindung auch mit der Zeit stärker wird (Bickmore et al. 2005). Teilnehmende, die den Chatbot Woebot nutzten, berichteten, dass sich der Chatbot einfühlsam, echt und sorgsam anfühlt (Fitzpatrick et al. 2017). Teilnehmende, die glaubten, dass sie mit einem virtuellen Agenten interagierten, berichteten von einem geringeren Widerstand, sich selbst zu öffnen, und einem geringeren Eindrucksmanagement als diejenigen, die glaubten, mit einem echten Menschen zu interagieren (Gratch et al. 2014). Im Setting der Künstlichen Intelligenz scheint der Aufbau einer Arbeitsbeziehung demnach kein größeres Problem darzustellen.

### Offene Fragen zur Arbeitsbeziehung im digitalen Coaching

Ebenso wie im traditionellen Präsenz-Coaching stellt sich auch im digitalen Coaching die Frage, wie die Arbeitsbeziehung konkret unterstützt werden kann. Hierbei mag es Unterschiede geben, da bei digitalen Kommunikationsmedien andere Hilfsmittel oder Interaktionen möglich sind, wie z. B. durch die Nutzung von virtuellen Avataren. Insbesondere beim konkreten Verhalten könnte es hier interessant sein, wie sich Coaches und Coachees in diesen virtuellen Welten verhalten und ob sich dies auf die Arbeitsbeziehung auswirkt. Da Coaching durch Künstliche Intelligenz wahrscheinlich zunehmen wird und auch grundsätzlich zu funktionieren scheint, stellt sich die Frage, warum und wie sich die Arbeitsbeziehung zu rein virtuellen Agenten konkret entwickelt. Bislang ist zu sehen, dass sie sich entwickelt, aber es bleibt unklar, ob es sich weiterhin um dasselbe Konstrukt Arbeitsbeziehung handelt, und wenn ja, aus welchen Gründen sie entsteht.

### Empfehlungen für die Coaching-Praxis

Da Coaching bereits durch die Covid-19 Pandemie vielfach mit digitalen Medien praktiziert wurde und die empirische Evidenz bislang ebenfalls dafür spricht, dass Coaching weiterhin wirksam ist, besteht grundsätzlich wenig Anlass zu Bedenken in Bezug auf die Gestaltung einer starken Arbeitsbeziehung. Da Passarelli et al. (2020) zeigen, dass die Selbstwirksamkeit im Umgang mit dem ausgewählten Medium entscheidend ist, sollten Coaches (und auch Coachees) keine Angst vor der Nutzung dieser Medien haben und sich mit ihnen vertraut machen. Dies gilt nicht nur für den Umgang mit Videokonferenz-Software, sondern auch für den Umgang mit virtuellen Avataren oder der Zusammenarbeit mit Chatbots, die in die eigene Praxis integriert werden können und vor allem zwischen den Sitzungen zur weiteren Reflexion anregen oder zum Monitoring dienen können. Ein weiteres Zukunftsszenario für das Coaching wird hier deutlich: Coaches benötigen mehr Designkompetenz, um den Coachingprozess an die Bedürfnisse der Coachees individuell anzupassen (Schermuly et al. 2021). Hierzu können insbesondere Coaching-Weiterbildungen und Verbände beitragen, um Coaches dabei zu unterstützen, sich darauf gezielt vorzubereiten.

## Limitationen

Trotz der mittlerweile erheblichen Forschungsbemühungen zur Arbeitsbeziehung im Coaching bestehen dennoch einige grundsätzliche Limitationen in diesem Forschungsbereich. Der Großteil der Schlussfolgerungen beruht auf nicht-experimentellen Daten und lässt somit keine Kausalität zu. Mittlerweile gibt es immer mehr Studien, die den Coachingprozess im Längsschnitt untersuchen, aber die Wirkrichtung wird letztendlich nur mit Hilfe von Experimenten möglich sein. Zudem stammen etliche Konzepte und Gedankengänge aus der älteren und reichhaltigeren Psychotherapieforschung und müssen im Anwendungsfall Coaching empirisch stärker untersucht werden. Dann wären zuverlässigere Schlussfolgerungen und auch sichere Handlungsempfehlungen für die Coaching-Praxis möglich. Darüber hinaus basieren viele der hier dargestellten Studien auf Fragebogen-Erhebungen. Insbesondere wenn es um tatsächliches Verhalten geht, wäre es hier wünschenswert, wenn die Erkenntnisse auf Verhaltensbeobachtungen basieren. Da Coaches und Coachees in ihrer Einschätzung der Arbeitsbeziehung durchaus abweichen können, wäre auch die gemeinsame Befragung beider Akteure von Nutzen, um hier Unterschiede feststellen zu können. Weiterhin wurde bislang kaum differenziert, um welches Coachingverständnis es sich in den zugrunde liegenden Coachingprozessen und Studien handelt. Den meisten der hier aufgeführten Studien liegt eher ein Verständnis von Coaching zugrunde, das auch leichter empirisch-quantitativ abgebildet werden kann. Eine differenzierte Betrachtung bei verschiedenen Coachingverständnissen wäre wünschenswert für die weitere Coachingforschung und -praxis.

Trotz dieser Limitationen bleibt die These plausibel, dass es sich bei der Arbeitsbeziehung um einen der zentralen Faktoren im Coaching handelt, sowohl für Coaches als auch Coachees. Sie trägt zur Erklärung bei, warum manche Coachingprozesse erfolgreicher verlaufen als andere. Weitere Forschungsarbeiten werden dazu beitragen, Coachingprozesse bestmöglich zu gestalten, sodass Coachees vom größtmöglichen Coachingerfolg profitieren können.
